# Assessment of patient safety culture among the staff of the University Hospital for Gynecology and Obstetrics in Alexandria, Egypt

**DOI:** 10.1186/s42506-022-00110-8

**Published:** 2022-10-12

**Authors:** Hend Mostafa Ali Ali, Asmaa Mahmoud Abdul-Aziz, Eman Ahmed Fawzy Darwish, Manal Shfik Swelem, Eman Anwar Sultan

**Affiliations:** 1 Community Medicine Department, Faculty of Medicine, Alexandria University, Alexandria, Egypt; 2 Gynecology and Obstetrics Department, Faculty of Medicine, Alexandria University, Alexandria, Egypt

**Keywords:** Patient safety culture, El-Shatby, Gynecology and Obstetrics, Alexandria

## Abstract

**Background:**

Patient safety (PS) is a fundamental component of healthcare quality. Patient Safety Culture (PSC) assessment provides an organization with insight of perceptions and attitudes of its staff related to patient safety. In addition, it is meant to improve performance rather than blaming individuals. This study aimed to assess patient safety culture from the health care staff perspective in El-Shatby University Hospital for Gynecology and Obstetrics.

**Methods:**

A descriptive cross-sectional study was conducted. The study was conducted at El-Shatby University Hospital for Gynecology and Obstetrics from November 2020 to January 2021. The target participants were assistant lecturers, residents, and head nurses in charge during the field study period. The number of potential participants who fulfilled the inclusion criteria (in charge during the period of data collection and working in the hospital for more than 3 months) was 83; the twelve participants who participated in the pilot study were excluded. The total number of participants who agreed to participate in the study was 66 participants (38 residents, 18 assistant lecturers, and 10 head nurses) out of 71 potential participants representing a 92.9% response rate. A structured self-administered questionnaire format adapted from Hospital Survey on Patient Safety Culture (HSOPSC) questionnaire was distributed anonymously to the participants. The questionnaire has 42 items measuring twelve patient safety culture dimensions: teamwork within the unit, supervisors’ expectations and actions to promote patient safety, feedback and communication about error, organizational learning, communication openness, overall perception of patient safety, hands-off and transitions, teamwork across units, frequency of events reported, management support for patient safety, staffing, and management support for patient safety. Except for two items that are responded on a five-point frequency scale (never, rarely, sometimes, most of the time, and always) the majority of patient safety culture questions are answered on a five-point agreement scale (strongly disagree, disagree, neutral, agree, and strongly agree), with a higher score indicating a more favorable attitude toward patient safety.

**Results:**

The overall average positive percent score was 45.4%. Average positive response percentages to individual items ranged from 28.8 to 81.8%. No domain had an average positive percent score of more than 75%. Out of the twelve dimensions of patient safety culture included in the HSOPSC questionnaire, “the teamwork within unit” domain had the highest average positive percent score (62.1%) among all participants. On the other hand, the “Non-punitive response to error” domain had the lowest score (18.9%). More than half (57.6%) of the participants rated patient’s safety at the hospital as acceptable.

**Conclusion:**

Investing in practices that strengthen patient safety is crucial if the hospital is to improve overall performance and quality of services. The present study displays a frail patient safety culture (PSC) in the majority of the domains. All the domains should be considered of high priority focused areas for remark and reformative tasks. Continuous training programs of the staff on patient safety to improve their perception of safety culture are necessary. All PSC composites need improvement starting with regular assessment of PSC along with continuous monitoring and increasing the healthcare providers’ awareness of demanded PSC.

## Introduction

Despite significant breakthroughs in technology and skills in health care over the last few decades, patients continue to be harmed by medical errors. In the year 2000, adverse events affected 3.7% of all hospitalized patients in the USA. The adverse events resulted in a longer stay in the hospital, a disability upon discharge, or both. The report “To Err is Human: Building a Safer Health System,” which indicated that medical errors were one of the major causes of death and injury in the United States, stimulated international interest in patient safety [1]. The world health organization (WHO) describes patient safety as the prevention of adverse events for patients. Error prevention, learning from errors, and building safety culture involving healthcare professionals and patients are important factors when healthcare aims towards patient safety improvement [2, 3]. Patient safety culture is an essential factor to consider when evaluating healthcare quality [4].

Many safety-oriented organizations develop and foster a patient safety culture which is defined by the WHO as “the shared values among organization members about what is important, their beliefs about how things work in the organization, and the interaction of these within work unit and organizational structures and systems, which together produce behavioral norms in the organization that promote safety.” (WHO, 2009) [5]. Patient safety culture assessments are required by international accreditation agencies. Such assessments are easiest to conduct through surveys that evaluate the perception of healthcare staff on many issues such as teamwork, management and leadership support to patient safety, staffing, incident reporting, and other issues pertaining to safety [6].

It is essential to examine a health care organization's current safety culture in order to build an effective safety culture. Information regarding staff’ safety-related beliefs and behavior helps identify areas of weakness and strength for designing and implementing interventions aimed at improving the safety culture [7]. In Egypt, different studies highlighted the need for improving the PSC among healthcare providers. A study conducted in Fayoum showed that overall, patient safety in Fayoum's public hospitals was poor. The total patient safety score was 46.56%. The highest mean composite score was for organizational learning and continuous improvement (65.36%) and the lowest reported score was for communication openness (17.9%) [8]. A study conducted in Alexandria University ICU concluded that the total composite percent positive score was 37.3%. The “Teamwork within Units” dimension had the utmost average percentage positive score (63.5%) among all participants, on the other hand, the “Non-Punitive Response to Errors” dimension had the lowest one (12.0%) [9]. Similar findings were reported in Alexandria primary healthcare services [10].

Worldwide, several studies were conducted to assess PSC with varying results. In Lebanon, a survey conducted in sixty-eight Lebanese hospitals (54% of the hospitals) found that the dimension with the highest positive ratings was teamwork within units (82.3%), while the dimension with the lowest ratings was a non-punitive response to error (24.3%) [11]. In the USA, the Hospital Survey on Patient Safety Culture (HSOPSC) user comparative database report published in 2014 concluded that the smallest hospitals (6–24 beds) had the highest percent positive average across all composites (69%), while larger hospitals (400 beds or more) had the lowest (61%). Non-teaching hospitals on average scored higher than teaching hospitals by 5 percentage points or more on 6 of the 12 composites [12].

Several studies evaluate the knowledge about patient safety culture among healthcare members and identify the critical areas of healthcare facility. However, data about patient safety culture in healthcare facilities providing services in the field of obstetrics and gynecology are insufficient. The identification of the negative & positive attitudes of the health care workers towards patient safety culture is the potential for improvement and planning action to corroborate and endure a commitment to safer care. The aim of the present study was to assess patient safety culture among the staff of El-Shatby University Hospital for Gynecology and Obstetrics.

## Methods

### Study design and setting

A descriptive cross-sectional study was conducted at El-Shatby University Hospital for Gynecology and Obstetrics over a 3-month period from November 2020 to January 2021. The hospital serves four governorates including Alexandria, El-Behira, Matrouh, and Kafr El-Sheikh. It has six departments, one ICU unit, and the eclampsia unit. The hospital has ten operating theatres: four for emergency operations, four for elective surgeries, and two for endoscopic surgeries. It provides both inpatient (254 beds) and outpatient (six outpatient clinics) services. The hospital has a mandatory error reporting system in which the reports received, analyzed, and feedback is provided to the reporter. The providers are held accountable for their mistakes.

### Inclusion criteria

A comprehensive sample of all staff members (in charge) during the study period, including assistant lecturers, residents, and head nurses, who were working in the hospital for more than 3 months, were enrolled in the study.

The total number of potential participants who fulfilled the inclusion criteria was 83; the twelve participants who participated in the pilot study were excluded. The total number of participants who agreed to participate in the study was 66 participants (38 residents, 18 assistant lecturers, and 10 head nurses) out of 71 potential participants representing a 92.9% response rate.

### Data collection

The questionnaire used to measure the patient safety culture was the adapted form of The Hospital Survey on Patient Safety Culture (HSOPSC) questionnaire [12] and the percentages of the positive responses of health care staff were assessed. The questionnaire was used in English and comprises two parts: the first one includes demographic characteristics, work position and years of working experience. The second part was PSC composites which comprised of 12 safety culture composites and a total number of 42 items. The twelve patient safety culture dimensions include; teamwork within the unit, supervisors expectations and actions to promote patient safety, feedback and communication about error, organizational learning, communication openness, overall perception of patient safety, hands-off and transitions, teamwork across units, frequency of events reported, management support for patient safety, staffing and management support for patient safety. Except for two items that are responded on a five-point frequency scale (never, rarely, sometimes, most of time, and always) the majority of patient safety culture questions are answered on a five-point agreement scale (strongly disagree, disagree, neutral, agree, and strongly agree), with a higher score indicating a more favorable attitude toward patient safety.

A preliminary phase was conducted to assess the validity and reliability of the adapted version of the questionnaire (where some modifications were made to the wording of the questions of the original questionnaire). For the assessment of content validity, three staff members experts in the field of public health were invited to assess the degree to which the items in the questionnaire were relevant and could correctly measure safety culture among the study participants and their remarks were taken into consideration. Test–retest reliability was measured by administering the questionnaire twice. Responses were collected 3 weeks apart. The test–retest reliability coefficient (Cohen’s kappa) was 0.97. Internal consistency was measured using inter-item correlation; Cronbach’s alpha = 0.78, while Cronbach’s alpha of the original questionnaire ranged from 0.63 to 0.84).

### Pilot study

A pilot study was conducted before starting the field work. A random sample (*n* = 12) from the study hospitals’ residents, assistant lecturers, and nurses were asked to answer the self-administered questionnaire and were excluded from the study sample. The results of the pilot study were one question was double negative, so it was re-phrased. The questions were properly understood by participants. The questionnaire took 15–20 min to be completed.

Data was collected at the workplace of the participants (emergency room, outpatient clinics, operation theatre, ultrasonography rooms, and inpatient wards). The self-administered questionnaire was distributed anonymously to the participants. The principle of patient safety culture and the aim of the study were explained to them, and they were asked to fill in the questionnaires. The participants completed the questionnaire while the investigator was available to respond to any issue. The questionnaire was filled in within an average of 15–20 min. Completeness of the questionnaires was checked at the spot.

### Statistical analysis

Data were coded, tabulated, and analyzed using (SPSS) version 25 [13]. Categorical data was expressed as numbers and percentages.

#### Calculation of percent positive scores [14]

Based on HSOPSC user’s database published in 2018 [14], HSOPC’s 42 items had been grouped into twelve domains. Each of the twelve patient safety culture domains is composed of three or four survey items. Composite scores for each domain were calculated by averaging the percent positive response on its items. Out of the twelve domains, nine domains ask respondents to answer using 5-point response categories in terms of agreement (strongly agree, agree, neither, disagree, and strongly disagree). The survey items of the remaining three domains (feedback and communication about error, communication openness, and frequency of events reports) use 5-point response categories in terms of frequency (always, most of the time, sometimes, rarely, and never).

The percent positive response is calculated as follows:

The agreeing with positively worded items takes a score 1 and disagreeing takes the score 0 and vice versa to the negatively worded items. The overall score for each dimension is calculated by adding the percentage of positive responses and then dividing them by the number of items in the dimension. For example, the domain “teamwork within the unit” had three items; people support one another (the positive response = 53.1%), when a lot of work needs to be done quickly, we work together as a team to get the work done (the positive response = 81.8%), and people treat each other with respect (the positive response = 51.5%). The overall score for the dimension = (53.1 + 81.8 + 51.5) divided by 3. So, the overall positive response of that dimension is 62.1%.

For positively worded items, the percent positive response is the combined percentage of respondents who answered “strongly agree” or “agree”/ “always,” or “most of the time”. For negatively worded items, the percent positive response is the combined percentage of respondents who answered “strongly disagree” or “disagree,”/ “never” or “rarely,” because a negative answer on a negatively worded item indicates a positive response.

#### Strengths and areas for improvement

Consequently, based on HSOPSC user comparative data report (2010) [15], the results are classified based on percent positive response into three categories:

- Areas of strengths: when the percent positive response is more than 75%.
- Areas with the potential for improvement: the percent positive response is 50–75%.
- Areas of weakness: the percent positive response is lower than 50%.

## Results

Out of the 71 potential participants who fulfilled the inclusion criteria 66 participants (38 residents, 18 assistant lecturers, and 10 head nurses) agreed to participate in the study with a response rate of 92.9%.

Table 1 shows that more than half of the surveyed participants were residents (57.6), while more than one-quarter of them were assistant lecturers (27.3%), and 15.1% were head nurses. More than half of them were males (56.1%). 63.6% of the participants were working from 1 to < 5 years, while 15.2% were engaged in work from 3 months to < 1 year. Nearly one-tenth (9.1%) were working for 5 to < 10 years and those who spent 10 years and more in work were 12.1%. The highest percentage (40.9%) of the study sample had 80 working hours and more per week. Just less than a third (30.3%) were working from 40 to < 60 h per week. Less than one-fifth (18.2%) had 20 to < 40 working hours per week and about a tenth (10.6%) of the participants were working for 60 to < 80 h per week.

**Table 1:** Background characteristics of the surveyed staff (*n* = 66) of El-Shatby University Hospital for Gynecology and obstetrics, Alexandria, Egypt, November 2020 to January 2021

Background characteristics	(*N* = 66)	%
**Job**		
Resident	38	57.6
Assistant lecturer	18	27.3
Head nurse	10	15.1
**Gender**		
Males	37	56.1
Females	29	43.9
**Duration of work in the current hospital (years)**		
3 months< 1 year	10	15.2
1–	42	63.6
5–	6	9.1
10 and more	8	12.1
**Working hours per week**		
20–	12	18.2
40–	20	30.3
60–	7	10.6
80+	27	40.9

Table 2 portrays the average percent positive score of the domains of patient safety culture. The total average percent positive score of the twelve domains was 45.4%, ranging from 18.9% to 62.1%. No domain had an average positive percent score of more than 75.0%. The safety culture domains with the highest average percent positive scores of more than 50.0% (areas with potential for improvement) were “teamwork within unit” (62.1%), followed by supervisors’ expectations and actions to promote patient safety” (58.7%),” feedback and communication about error” (56.1%), “organizational learning and continuous improvement “(56.1%), and “communication openness” (54.5%). Seven out of the twelve domains represent areas of weakness (average percent positive scores less than 50.0%) include; “overall perception of patient safety” (49.9%) “hands off and transitions” (41.6%), “teamwork across units” (41.6%), “frequency of events reported” (39.3%), management support for patient safety (36.4%), “Staffing” (29.5%). The domain with the least average percent positive score is “Non-punitive response to error” (18.9%).

**Table 2:** Distribution of the surveyed staff (*n* = 66) of El-Shatby University Hospital for Gynecology and obstetrics during the study period (November 2020 to January 2021) by their response on Patient Safety Culture items and domains

Domain	Average positive response %	Item	Positive response frequency (*n* = 66)	Positive response %
**1. Teamwork within unit**	**62.1**	a. People support one another	35	53.1
		b. When a lot of work needs to be done quickly, we work together as a team to get the work done	56	81.8
		c. People treat each other with respect	34	51.5
**2. Supervisors’ expectations and actions to promote patient safety**	**58.7**	a. My supervisor/ manager says a good word when he/she sees a job done according to established patient safety procedures	41	62.1
		b. My supervisor/manager seriously considers staff suggestions for improving patient safety	47	71.2
		c. Whenever pressure builds up, my supervisor/manager wants us to work faster even if it means taking shortcuts. ^a^	29	28.8
**3. Feedback and communication about error**	**56.1**	a. We are given feedback about changes put into place based on event reports	28	42.5
		b. We are informed about errors that happen in this unit	38	57.6
		c. In this unit, we discuss ways to prevent errors from happening again	45	68.2
**4. Organizational learning—continuous improvement**	**56.1**	a. We are actively doing things to improve patient safety	50	75.7
		b. Mistakes have led to positive changes here	32	48.5
		c. After we make changes to improve patient safety, we evaluate their effectiveness	41	62.2
**5. Communication ppenness**	**54.5**	a. Staff will freely speak up if they see something that may negatively affect patient care	36	54.5
**6. Overall perceptions of patient safety**	**49.9**	a. It is just by chance that more serious mistakes don’t happen around here. ^a^	33	50.0
		b. Patient safety is never sacrificed to get more work done	39	59.0
		c. We have patient safety problems in this unit. ^a^	22	33.3
		d. Our procedures and systems are good at preventing errors from happening	38	57.6
**7. Handoffs and transitions**	**41.6**	a. Important patient care information is often lost during shift changes. ^a^	33	50.0
		b. Problems often occur in the exchange of information across hospital units. ^a^	22	33.3
**8. Teamwork across units**	**41.6**	a. There is good cooperation among hospital units that need to work together	27	40.9
		b. It is often unpleasant to work with staff from other hospital units. ^a^	28	42.4
**9. Frequency of events reported**	**36.4**	a. When a mistake is made, but is caught and corrected before affecting the patient, how often is this reported?	29	43.9
		b. When a mistake is made, but has no potential to harm the patient, how often is this reported?	25	37.8
		c. When a mistake is made that could harm the patient, but does not, how often is this reported?	24	36.3
**10. Management support for patient safety**	**39.3**	a. Hospital management provides a work climate that promotes patient safety	22	33.4
		b. Hospital management seems interested in patient safety only after an adverse event happens.^a^	26	39.4
**11. Staffing**	**29.5**	a. We have enough staff to handle the workload	28	42.4
		b. We work in “crisis mode” trying to do too much, too quickly. ^a^	11	16.6
**12. Non-punitive response to error**	**18.9**	a. Staff feel like their mistakes are held against them. ^a^	6	9.1
		b. Staff worry that mistakes they make are kept in their personnel file. ^a^	19	28.8
**Total average percent positive score**	**45.4**			

^a^Indicates a negatively worded item, where the percent positive response is based on those who responded “strongly disagree” or “disagree, or neutral” or “never” or “rarely” or “sometimes” (depending on the response category used for the item)

**Fig. 1:**
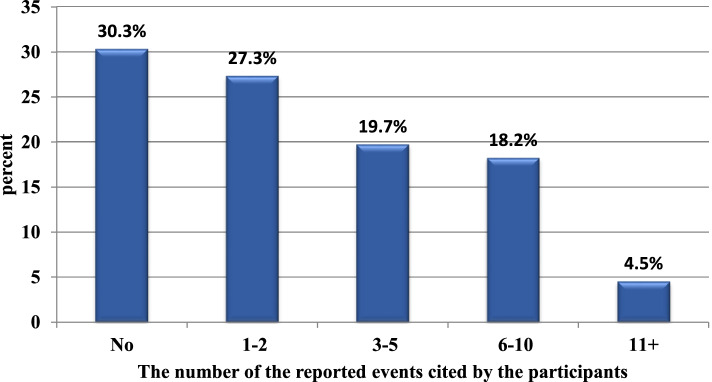
The number of the reported events cited by the participants at the University Hospital for Gynecology and Obstetrics, Alexandria, Egypt, November 2020 to January 2021

Figure 1 illustrates the number of reported events cited by the participants during the past twelve months. Less than one-third (30.3%) of the participants did not report any event, while 69.7% reported from one to more than 11 event reports during the past twelve months.

**Fig. 2:**
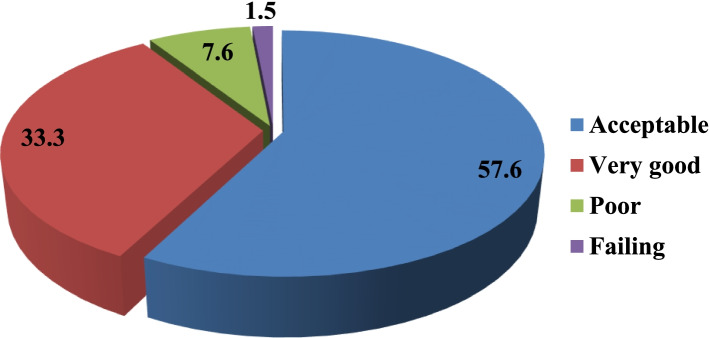
Participants’ grading of patient safety within the working unit, University Hospital for Gynecology and Obstetrics, Alexandria, Egypt, November 2020 to January 2021

Figure 2 demonstrates that many of the surveyed participants (57.6%) perceived that their working unit had an acceptable patient safety grade. Those who regard working unit as good, poor, and failing were 33.3, 7.6, and 1.5% respectively.

## Discussion

Patient safety is a fundamental component of healthcare quality [16]. Safety culture assessment provides an organization with insight of perceptions and attitudes of both managers and staff related to patient safety. Furthermore, it is meant to improve performance rather than condemning individuals [17].

The present study revealed that the overall average percent score of safety culture is 45.4% and the total average percent positive score of the individual items ranged from 16.6 to 81.8%. This was matched with the results of a study conducted by El-Shabrawy et al. in 2015 in Beni –Suef University Hospital [18], who reported a score of 40%. A similar score was reported in a study conducted by El-Sherbiny et al. in El-Fayoum governorate (2020), with a score of 46.5% [8]. However, it was lower than the mean percent score of teaching hospitals in the USA (62.0%) in 2014 [15], and lower than scores reported by studies in Arab countries such as Lebanon 2010 [11] with a score of 61.5% and KSA 2010 [19] with a score of 61.0%. Higher scores of 65.0%, 64.0%, and 62.0 were also, reported in studies in China in 2013 [4] and Taiwan in 2010 [20], respectively. A possible explanation for these differences in results is the low awareness of patient safety culture. Moreover, the literature shows that safety culture differs across hospital organizations depending on the organization’s experience, size, and function [21]. Moreover, the presence or lack of elements encouraging positive PSC, such as a blame and shame culture in dealing with adverse events, open communication, and management support, could explain the discrepancy in the overall median percent score for perception of PSC in different settings [22]. In addition, a discrepancy in the results between different settings may be attributed to different characteristics of the participants and settings.

According to the present study, no domain had an average positive percent score of more than 75.0%. The safety culture domain with the highest average percent positive scores was “teamwork within unit” (62.1%), as the participants work together as a team when a lot of work needs to be done. Teamwork is reflecting the degree of collaboration, cooperation, and mutual respect among personnel working in the same environment. However, this study reported a lower average positive score (41.6%) for the domain teamwork across unit. This indicates poor communication and cooperation between units that results in misunderstanding and loss of information. In contrast, according to the World Health Organization's comparative database report (2008) in the Eastern Mediterranean Region, teamwork within units was area of strength for most hospitals [23]. This is a critical issue because patient care in hospitals is typically coordinated across multiple hospital units. Training on teamwork skills is recommended to strengthen teamwork within and across the units. Team training was found to be an effective strategy in improving safety culture [24].

On the other hand, the non-punitive response to error domain was the domain with the least positive percent score (18.9%). It reflects “blame and shame” culture in which failure is penalized or hidden and people refuse to admit that issues exist [25]. This result agrees with the results of a study conducted in Cairo in 2019 which revealed that the domain with the highest positive responses was; teamwork within units (92.2%) while the lowest positive response was the domain” non-punitive response to error” (20.8%) [26].

The results of the present study show a weak patient safety culture in the majority of the domains. The study identified seven domains that represent areas of weakness (average percent positive scores less than 50.0%). These are “overall perception of patient safety,” “hands off and transitions,” “teamwork across units,” “frequency of events reported,” “management support for patient safety,” “staffing,” and”non-punitive response to error.” These should be considered of high priority focused areas for remark and reformative tasks. Continuous training programs of the staff on patient safety to improve their perception of safety culture are needed.

The present study indicated that none of the participants rated the working unit as “excellent” in patient safety while 57.6% of them perceived it as an acceptable and 33.3% rated it as very good. This was similar to the percentage reported by the study conducted in Cairo in 2019 (48.7% and 30.0% respectively) [26].

Reporting errors seems to be a process that is often avoided. Efforts to identify mistakes may be undervalued in the Egyptian culture. This study revealed that about one-third (30.3%) did not report any event within the past 12 months; this is comparable to the study conducted in Cairo (2019) (32.7%) [26]. This may be explained by the tendency for closed culture as regards adverse events reporting in hospitals indicating underestimation of event rate [27]. Many medical errors go unreported, for a variety of reasons including fear, shame, the presence of a punitive response to errors, and the reality that reporting would not frequently result in definite change. In comparison to the United States, "fear of consequences" is more prominent in East Asia and the Middle East. Workplace climate/culture, on the other hand, is more frequently cited as a barrier in centers around the United States. Within the same country, reported barriers differed from one facility to another. These variances are most likely due to disparities in management strategies, reporting systems, work environment culture, and whether or not patient safety is a priority for hospital administration [28].

Handoff and transitions domain had a low score (49.9%); this indicates that problems often occur in the exchange of information across hospital units which consequently could adversely affect patient safety. It may be good to reschedule shifts and reorganize the staff. Adding handoff forms, handing off in verbal and written format is recommended [18].

Furthermore, staffing (29.5%) also seemed to be a challenge to participants in this study, as they indicated that there is not enough staff to handle the workload and that they work in crisis mode trying to do too much too quickly. Given the enormous body of research linking the availability of health care providers to population health outcomes, this conclusion is critical [29]. Organizations with limited staffing have experienced major patient-related disasters [30]. When the number of personnel needed to provide patient care is fewer than ideal, most employees are overworked, burned out, stressed, and sleepless, which can lead to gaps in performance, affecting quality and patient outcomes [30, 31]. Calculating the workload and providing adequate staff based on the calculated workload would be a solution to improve this domain.

According to the present study, the five safety culture domains that are potential for improvement (average percent positive scores more than 50.0%) were “teamwork within unit,” followed by “supervisors expectations and actions to promote patient safety,” “feedback and communication about error,” “organizational learning and continuous improvement,” and “communication openness.” This means that these dimensions need attention and corrective actions.

### Limitations of the study

Although the present study covered a large number of the staff of El-Shatby University Hospital for Obstetrics and Gynecology, one of its limitations is being restricted to one university hospital in Alexandria out of a total of twelve university hospitals. Larger scale studies including more hospitals to form a holistic picture of the situation in Alexandria and to create a database of patient safety culture in Alexandria university hospitals are needed.

## Conclusions

Investing in practices that strengthen patient safety is crucial if the hospital is to improve the overall performance and quality of services. The present study displays a frail patient safety culture (PSC) in the majority of the domains. No domain had an average positive percent score of more than 75.0%. All the domains should be considered of high priority focused areas for remark and reformative tasks. Continuous training programs of the staff on patient safety to improve their perception of safety culture are necessary. All PSC composites need improvement starting with regular assessment of PSC along with continuous monitoring and increasing the healthcare providers’ awareness of demanded PSC. Continuous training of health care workers on skills supporting PSC is strongly recommended along with the implementation of proactive risk management that focuses on the errors in the system or process, rather than the individual’s fault. A blame-free environment should be created to detect threats to patient safety, share information, and learn from events.

## Data Availability

The dataset used and analyzed during the current study is available from the corresponding author on reasonable request.

## Abbreviations

HSOPSC: Hospital Survey On Patient Safety Culture
PS: Patient safety
PSC: Patient safety culture
AEs: Adverse events
WHO: World Health Organization
HCWs: Healthcare workers
SPSS: Statistical Package for Social Science
